# Novel Treatment of Chronic Bladder Pain Syndrome and Other Pelvic Pain Disorders by OnabotulinumtoxinA Injection

**DOI:** 10.3390/toxins7062232

**Published:** 2015-06-18

**Authors:** Jia-Fong Jhang, Hann-Chorng Kuo

**Affiliations:** Department of Urology, Buddhist Tzu Chi General Hospital, and Tzu Chi University, Hualien 970, Taiwan; E-Mail: alur1984@hotmail.com

**Keywords:** interstitial cystitis, chronic prostatitis, pelvic floor

## Abstract

Chronic pelvic pain (CPP) is defined as pain in the pelvic organs and related structures of at least 6 months’ duration. The pathophysiology of CPP is uncertain, and its treatment presents challenges. Botulinum toxin A (BoNT-A), known for its antinociceptive, anti-inflammatory, and muscle relaxant activity, has been used recently to treat refractory CPP with promising results. In patients with interstitial cystitis/bladder pain syndrome, most studies suggest intravesical BoNT-A injection reduces bladder pain and increases bladder capacity. Repeated BoNT-A injection is also effective and reduces inflammation in the bladder. Intraprostatic BoNT-A injection could significantly improve prostate pain and urinary frequency in the patients with chronic prostatitis/chronic pelvic pain syndrome. Animal studies also suggest BoNT-A injection in the prostate decreases inflammation in the prostate. Patients with CPP due to pelvic muscle pain and spasm also benefit from localized BoNT-A injections. BoNT-A injection in the pelvic floor muscle improves dyspareunia and decreases pelvic floor pressure. Preliminary studies show intravesical BoNT-A injection is useful in inflammatory bladder diseases such as chemical cystitis, radiation cystitis, and ketamine related cystitis. Dysuria is the most common adverse effect after BoNT-A injection. Very few patients develop acute urinary retention after treatment.

## 1. Introduction

Chronic pelvic pain (CPP) is defined as chronic or persistent pain perceived in the structures related to all organs in the pelvis for at least 6 months’ duration, including prostate, scrotum, urethra, bladder, vagina, rectum, and anus [1].

The diagnosis of CPP is based on a patient’s history and physical examination. Then, patients are classified according to organ-specific symptoms. The European Urology Association guidelines classify patients with CPP according to the region where pain occurs (with or without a particular disease), pain system (urological, gynecological or musculoskeletal), and end-organ. Pain syndrome as identified from the history or examination (such as bladder, prostate, vulvar, or pelvic floor muscle). Many well-known diseases involve different pelvic organs. Chronic prostatitis/chronic pelvic pain syndrome (CP/CPPS), interstitial cystitis/bladder pain syndrome (IC/BPS), chemical cystitis, and pelvic floor muscle pain are categorized into CPP [1].

The pathogenesis of CPP is unclear. The CPP mechanisms involve ongoing acute pain mechanisms (such as those associated with inflammation or infection) and chronic pain mechanisms (such as central sensitization or visceral hyperalgesia) [1]. The EAU guidelines suggest the first CPP treatment should be simple analgesics, such as non-steroidal anti-inflammatory agents [1]. If the use of simple analgesics fails to provide adequate benefit, then opioids or neuropathic agents, such as gabapentin or amitriptyline, should be considered [1]. If the patient had an inadequate response to pain treatment, a specialist in pain management should be involved to help treat the patient’s pelvic pain.

Botulinum toxin (BoNT), which is produced by the bacterium *Clostridium botulinum*, is one of the most powerful neurotoxins and inhibits the release of the neurotransmitter acetylcholine from nerve fibers, thereby inhibiting muscle contractions [2]. Scott first used BoNT type A (BoNT-A) injected into human eye muscles to correct strabismus in 1981 [3]. Since then, BoNT-A has been widely used to treat many dystonic diseases and neuropathic pain syndromes. These conditions include cervical dystonia, cerebral palsy, trigeminal neuralgia, chronic migraine, and complex regional pain syndrome [4,5,6]. Recently, BoNT-A has been used to treat lower urinary tract diseases (LUTDs) including detrusor overactivity and benign prostate hyperplasia [7,8]. In addition, BoNT-A effectively treats CPP, IC/BPS, and pelvic floor muscle pain [9]. For patients with refractory CPP, BoNT-A has promising effects, including antinociceptive, muscle relaxation, and anti-inflammatory activity. This article reviews and analyses the current medical evidence on BoNT-A for treating CPP and the BoNT-A mechanism of action on painful LUTDs.

## 2. History of BoNT-A in LUTDs

The history of BoNT could be trace back to 18th century and it was first purified in 1928 [10,11]. Research on the therapeutic use of BoNT-A started as early as the 1960s. It has been used for treating LUTDs since the early 1990s.The first application of BoNT-A in LUTD targeted the urethral sphincter. Dykstra *et al.* reported his study of percutaneous or cystoscopic injection of the urethral sphincter in patients with spinal cord injury (SCI) and detrusor-sphincter dyssynergia in 1988 [12]. The urethral pressure and post-void residual urine (PVR) volume significantly decreased in these SCI patients. Studies of BoNT-A injection in the urinary bladder started in the early 2000s. Schurch *et al*. injected 200 U to 300 U of onabotulinumtoxinA (Allergan, Irvine, CA, USA) into the detrusor muscle during cystoscopy to treat detrusor hyperreflexia in SCI patients [13]. These patients resumed urinary continence with increased maximum cystometric bladder capacity. Many studies have been conducted to prove the efficacy of BoNT-A in detrusor overactivity, either neurogenic or non-neurogenic [14,15].

United States Food and Drug Administration approved the use of intravesical injection of onabotulinumtoxinA for neurogenic urinary incontinence and non-neurogenic overactive bladder in 2011 and 2013, respectively [16,17]. Intravesical Botox injection had been proven to be effective in neurogenic and idiopathic detrusor overactivity [18]. Investigating Botox-induced effects in patients with neurogenic detrusor overactivity, Conte and Giannantoni neurophysiologically demonstrated that Botox modulates bladder afferent pathways. Recent evidence also showed that Botox decreases nerve growth factor (NGF) in bladder tissue [19,20]. Since 2005, BoNT-A has been used to treat urological conditions of CPP. Smith *et al*. first treated female patients with IC/BPS, who received 100 U to 200 U of onabotulinumtoxinA with cystoscopic hydrodistention at the same time [21]. For such a disease without a definitively effective treatment, the study showed promising results and suggested that BoNT-A had an antinociceptive effect on bladder afferent pathways in patients with IC/BPS. This result encouraged and revolutionized the treatment of CPP. As recently as 2010, researchers began investigating BoNT-A for the treatment of CPP related to different conditions including myofascial pain, chemical cystitis, and CP/CPPS [22,23,24].

## 3. Mechanism Action of BoNT-A on CPP

**Figure 1:**
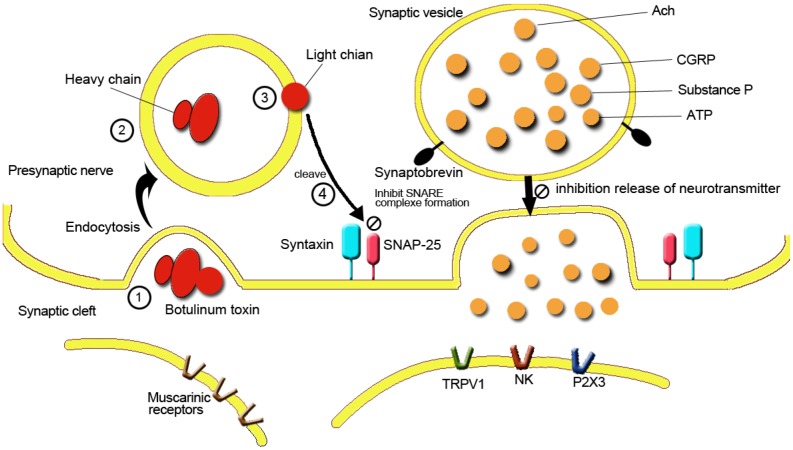
Mechanisms on BoNT-A inhibition of neurotransmitters release.

The possible mechanism of BoNT-A act on CPP is illustrated on Figure 1. In both animals and humans experimental studies of pain it has been observed that up-regulation of membranes receptors/channels in the nociceptors, such as the TRPV1 and P2X3 may directly contribute to the neuropathic pain [25,26]. TRPV1 has been shown to be expressed in small diameters sensory fibers, some of which contain various neuropeptides such as SP and CGRP [27]. TRPV1 is also found in nervous central tissue and non neuronal tissues where it exhibits functions related to hyperalgesia. The expression of TRPV1 has been shown to be up-regulated during nerve injury induced thermal hyperalgesia [27]. In addition, BoNT/A has been observed to reduce hyperalgesia and TRPV1 expression in the DRG neurons in rats with neuropathic pain consequent to ventral rooth transection [28]. NGF has been shown to play a role in urinary dysfunction and pain [29]. Recent studies also found increasing serum and urinary NGF in the IC/BPS patients and it had been considered as a useful biomarker for the diagnosis of IC/BPS [30,31]. In particular, a randomized, double-blind, placebo controlled phase 2 study reported the anti-NGF antibody tanezumab could improve pain in patients with IC/BPS, suggesting that an NGF-sensitive pathway is involved in the bladder pain experienced by these patients [32].

### 3.1. Effectiveness of BoNT-A in the Treatment of Pelvic Pain

BoNT-A relieves pelvic pain via three primary mechanisms acting on the muscles, central nervous system (CNS), and inflammation.

### 3.2. Reducing Pelvic Muscle Spasm and Pain

Tenderness of pelvic muscles was highly prevalent among women with CPP, and spasm of the pelvic floor muscles is conventionally believed to be associated with CPP [33,34]. Perhaps the most straightforward explanation of how BoNT-A can be effective in the treatment of pain is a consideration of its direct impact on “muscle pain”. Muscles have a rich array of nociceptors, including thinly myelinated group III afferents and unmyelinated group IV fibers [35]. Muscle pain can result from ischemic, chemical, or mechanical stimulation. Chemicals known to sensitize muscle pain receptors is extensive and includes bradykinins, prostaglandin E, and a variety of neuropeptides, such as substance P and calcitonin gene-related peptide (CGRP) [35]. Adenosine triphosphate (ATP) also can act as a peripheral pain mediator and is present in large amount in muscle [36]. Results of animal studies show that BoNT-A blocks the release of noxious neurotransmitters including calcitonin CGRP, glutamate, adenosine triphosphate ATP, and substance P from neurons [37,38,39,40,41]. BoNT could block these neurotransitters of muscle nociceptors, and reduce muscle-related pain in the patients with CPP [35]. In addition, BoNT could blocks muscle contraction by alpha and gamma motor neurons and inhibit pelvic floor muscle spasm, leading to relief the pain induced by muscle spasm [35].

### 3.3. Reduction of Central and Peripheral Nerve Sensitization and Decrease of Noxious Neurotransmitter Release

Central sensitization is a process of the CNS in which the responsiveness of the central neurons to input from peripheral receptors is increased [42]. Recent studies suggest CPP patients may have hyperexcitability of the CNS [43]. In addition, CPP may involve an increase in noxious stimulus transmission [43]. As above-mentioned, BoNT-A could block neurotransmitters including substance P, CGRP and ATP, and then inhibit activation of neurons in the spinal cord responsible for the transmission of pain signals [37,38,39,40,41,42]. To date there is the evidence that BoNT-A antinociceptive effect is centrally mediated. Recent studies revealed that peripheral injection of BoNT-A reaches the CNS by retrograde transport, and BoNT-A has antinociceptive activity via CNS regulation [43,44]. In addition, current evidence suggested peripheral injection of BoNT-A could block CNS synaptic transmission including glutamate, dopamine, ATP, gamma-aminobutyric acid (GABA) [45]. Central antinociceptive action of BoNT-A also is associated with the activity of endogenous opioid system involving central µ-opioid receptors [46]. An experimental study suggested that BoNT-A acts synergistically with morphine and may counter act the tolerance associated with use of high doses of opioids [46]. Another important issue is clinical significance of axonal transport of BoNT-A in neurons is not clear. Up to now, evidence clearly showed BoNT-A can undergo anterograde axonal transport and transcytosis in neurons. Restani *et al.* reported significant levels of BoNT-A-cleaved synaptosomal-associated protein appeared in retina after BoNT-A injection in rat eye [47]. When BoNT-A applicated in peripheral organs, it might be axonally transported to brain sensory and motor regions, depending on the innervation of injected sites. A recent study showed increased technetium-99m in lumbosacral dorsal root ganglia after intravesical radiolabelled BoNT-A injection [48].

### 3.4. Reducing Neurogenic Inflammation

Neurogenic chronic inflammation of the pelvic organs is also a possible mechanism of CPP. Current evidence suggested persisted chronic inflammation contributes to IC/BPS, CP/CPPS, and pelvic floor muscle pain [49,50,51]. Increased mast cells, increased cytokine expression of interleukin 6, 10, 17, prostaglandin and NGF could be found in IC/BPS and CP/CPPS [52,53,54]. All of these could trigger vasodilatation, leakage of plasma, mast cell degranulation, and the lead to inflammation in tissue [49,50]. The inflammatory cytokines in the pelvic organs might be the key mechanism that induces pain in the patients with CPP [51]. In adddtion, the inflammation in pelvic organs also might lead to the others pathologic changes. For example, urothelium barrier dysfunction and cell apoptosis in IC/BPS might be attributed to the persisted inflammation in bladder [52]. BoNT-A injection could normalize inflammatory cytokines release including prostaglandin E2, substance P, nitric oxide and CGRP in peripheral tissue [41,43,55,56,57,58]. BoNT-A appears to have an anti-inflammatory effect on CPP, by blocking abnormal cytokine release in pelvic organ, and then relief pain in these patients.

The mechanisms of BoNT-A acted on neurogenic detrusor overactivity and CPP have some similarity and some difference. BoNT-A injection normalized the neurotransmitters release such as acetylcholine, ATP, substance P and CGRP. Patients with neurogenic detrusor overactivity could decrease abnormal detrusor contraction by BoNT-A direct action of the blocking acetylcholine and ATP. In contrast, the CPP patients might have benefit from more complicated mechanisms which involving more neurotransmitters as mentioned above.

## 4. Clinical Application of BoNT-A in IC/BPS

IC/BPS is a distressing syndrome of chronic bladder pain and is diagnosed on the basis of chronic (>6 months) pelvic pain, pressure or discomfort perceived to be related to the bladder, and at least one other urinary symptom such as persistent urge to void or urinary frequency [39]. The pathophysiology of IC/BPS is still unclear and is likely to be complex and multifactorial. Studies suggest several possible pathophysiological mechanisms, including urothelial dysfunction, abnormal activation of mast cells and central sensitization [59,60,61]. Many treatments, based on different possible pathophysiological characteristics, have been used to treat IC/BPS. These treatments include lifestyle modification, pain control medication, oral pentosan polysulfate, intravesical installation of dimethyl sulfoxide, and cystoscopic hydrodistention under general anesthesia [62]. However, about 30% to 40% of IC/BPS patients do not improve with these treatments. For IC/BPS refractory to conventional treatment, intravesical injection of 100 U to 200 U of onabotulinumtoxinA in the trigone and bladder floor, with cystoscopic hydrodistention, was first reported in 2004 in 13 patients [21]. The Interstitial Cystitis Symptom Index (ICSI) and Interstitial Cystitis Problem Index (ICPI) mean scores improved in these patients by 71% and 69%, respectively (*p* < 0.05). Urinary frequency, nocturia, and bladder pain (on a visual analog scale, VAS) also showed significant improvements. The onset of symptom relief was 5–7 days after treatment, and it persisted for an average of 3.7 months. In 2006, Giannantoni reported on 20 IC/BPS patients who received 200 U of onabotulinumtoxinA injected into the trigone and bladder floor [63]. Of them, 85% reported subjective improvements 1 month after therapy. The mean VAS pain scores, urinary frequency, and cystometric bladder capacity (CBC) improved significantly compared with the pretreatment values (all *p* < 0.05).

In a prospective, randomized, and controlled study, suburothelial injection with 100 U or 200 U of onabotulinumtoxinA and cystoscopic hydrodistention was compared with cystoscopic hydrodistention alone in patients with refractory IC/BPS [64]. In the 3-month follow-up period, the ICPI and ICSI significantly decreased in all three groups, but VAS reduction and CBC increases were significant only in the onabotulinumtoxinA injection groups [64]. A prospective study that enrolled 67 patients is currently largest series of onabotulinumtoxinA single injection in refractory IC/BPS in 2012 [65]. The 6-month follow-up results also showed significant improvements in ICPI, ICSI, VAS, and functional bladder capacity (FBC) (all *p* < 0.01). In a recent prospective, randomized, double-blind, placebo-controlled study of 60 refractory IC/BPS patients, intravesical supratrigone injection of 100 U of onabotulinumtoxinA at 20 sites was compared with normal saline injection at 20 sites with cystoscopic hydrodistention. Both groups showed improvements in ICPI and ICSI, but only the onabotulinumtoxinA group had a decrease of pain on the VAS pain scale and an increase in FBC. The improvement in the VAS pain scale was significantly better in the onabotulinumtoxinA group than in the normal saline group [66].

The long-term therapeutic effects of onabotulinumtoxinA are not achieved after a single injection. In a study of 15 IC/BPS patients receiving an injection with onabotulinumtoxinA 200 U, 13 patients (86.6%) reported symptom improvement at the 1 and 3-month follow-up evaluations [67]. However, the beneficial effects only persisted in 26.6% of patients at the 5- month follow-up evaluation. Bladder pain had recurred in all patients 12 months after treatment. Recent studies also suggest that symptomatic improvement after repeated onabotulinumtoxinA injection provided better long-term success rates than a single injection for the treatment of IC/BPS [68,69].

The therapeutic effect of onabotulinumtoxinA injection on the ulcerative type of IC/BPS is controversial. OnabotulinumtoxinA injection in 15 patients with ulcerative IC/BPS showed no significant change in any clinical or urodynamic variable after four sets of injections [70]. However, in another series of onabotulinumtoxinA treatments, trigonal injection in 10 patients with ulcerative IC/BPS and 14 patients with non-ulcerative IC/PBS showed significant improvements in pain intensity, urinary frequency, ICPI, and ICSI [71]. The discrepancy of therapeutic results might be due to different definition of ulcerative IC.

Although early studies also used 200 U of onabotulinumtoxinA in IC/BPS patients, most recent studies only used 100 U and had good therapeutic effects. Clinical studies of onabotulinumtoxinA for IC/BPS are summarized in Table 1. The injection site and the number of injections are controversial. OnabotulinumtoxinA injections in the trigone alone, both the trigone and the posterior bladder wall, and injections sparing the trigone are reported in the different studies. The injection number varied from 10 to 40 sites. Currently, no evidence suggests which treatment is best.

**Table 1:** Clinical studies for the use of onabotulinumtoxin Ain interstitial cystitis/bladder pain syndrome.

Authors, year	*N*	Follow-up	BoNT-A preparation, dose	Injection sites and volume	Frequency, Δ%	VAS, Δ%	ICPI, ICSI, Δ%	LoE	Others
Smith *et al.* 2004 [21]	13	3 mo	100 U to 200 U, 10 to 20 mL	20 to 30 sites, trigone and bladder floor	−44% *	−79% *	−69,−71% *	3	-
Giannantoni *et al.* 2006 [63]	14	3 mo	200 U, 20 mL,	20 sites, bladder floor and trigone	−35% *	−34% *	-	3	-
Giannantoni *et al*. 2008 [67]	15	3 mo	200 U, 20 mL,	20 sites, bladder floor and trigone	−43% *	−28% *	-	3	-
Kuo and Chancellor 2009 [64]	15	3 mo	200 U 20 mL + HD 100 U 20 mL + HD HDonly	40 sites, bladder floor except trigone	−34% *	−55% *	−42, −36% *	2	-
	29				−25%	−39% *	−38, −35% *		
	23				−14%	−18%	−23, −23% *		
Chung *et al*. 2012 [65]	67	6 mo	100 U, 20 mL	40 sites, bladder floor except trigone	−31% *	−37% *	−38, −34% *	3	-
Kuo, 2013 [69]	81	12 mo	100 U, 20 mL, 1 injection	40 sites, bladder floor except trigone	−23% *	−30% *	−28,−27% *	3	Repeat injection better than single injection
	30		100 U, 20 mL, 4 injections		−21% *	−37% *			
Lee *et al*. 2013 [70]	10 ulcer	6 mo	100 U, 20 mL, 4 injection	40 sites, bladder floor except trigone	0%	−10%	0%, −8%	2	BoNT-A injection is not effective in ulcer IC/BPS
	30 non ulcer		100 U, 20 mL, 4 injection		−68 *	−62% *	−65% *, −54% *		
Pinto *et al*. 2014 [71]	10 ulcer	1 mo	100 U, 10 mL	10 sites, trigone only	−29% *	−54% *	−46% *, −40% *	2	BoNT-A injection is effective in ulcer IC/BPS
	14 non ulcer		100 U, 10 mL		−23% *	−57% *	−47% *, −45% *		
Kuo *et al*. 2015 [66]	40	2 mo	100 U, 10 mL, +HD	20 sites, bladder floor except trigone	−27% *	−49% *†	−40% *, −34% *	1	Randomized study
	20		Normal saline 10 mL +HD		−9%	−24%	−30% *, 21% *		

Δ%: change from baseline, percentage. * Significant improvement for baseline, †: significant difference between different groups; HD: cystoscopic hydrodistention, mo: months, BoNT-A: botulinum toxin A.

Laboratory evidence shows the peripheral desensitization and anti- inflammatory effects of onabotulinumtoxin Ainjections in IC/BPS patients. In 19 IC/BPS patients who received intravesical onabotulinumtoxinA injections compared with 12 healthy controls [72], the baseline nerve growth factor (NGF) mRNA levels in the bladders of IC/BPS patients were significantly greater than in the control patients. After onabotulinumtoxinA treatment, the NGF mRNA levels decreased and were no longer significantly different from those of the controls. Immunohistochemical studies of NGF in bladder tissues also showed similar results. Urine NGF and brain-derived neurotrophic factor in IC/BPS patients significantly decreased 1 and 3 months after onabotulinumtoxinA injection [73]. Immunohistochemical studies of bladder specimens of IC/BPS patients showed significantly decreased tryptase, Bax, and apoptotic cell counts after repeat onabotulinumtoxinA injections [74]. This result suggests that BoNT-A injections improved chronic bladder inflammation and apoptosis in IC/BPS patients. Vascular endothelial growth factor (VEGF) in the bladder tissues of IC/BPS patients also decreased significantly after repeated onabotulinumtoxinA injections [75]. VEGF stimulates angiogenesis and is a mediator of inflammation [76]. The decrease of VEGF in the bladder tissues suggests subsidence of inflammation in the patients with IC/BPS.

In summary, many different clinical and laboratory studies show the effectiveness of onabotulinumtoxinA injections for the treatment of IC/BPS. However, the therapeutic duration and long-term follow-up results need to be investigated in larger, prospective studies. The long-term follow-up investigation of immunochemical staining of bladder specimens is also necessary.

## 5. Clinical Application of BoNT-A in CP/CPPS

Chronic pain in the region of the prostate is linked to the term “prostatitis” in male patients. However, only 10% of such cases have a proven bacterial infection [77]. The United States National Institutes of Health consensus classifies male patients with prostatitis syndromes into four categories. Patients without evidence of bacterial infection, active urethritis, urethral cancer, or stricture are assigned to category III CP/CPPS [78]. Currently, CP/CPPS is a distinct clinical entity defined as urologic pain or discomfort in the prostate region associated with urinary symptoms and/or sexual dysfunction lasting for at least 3 of the previous 6 months [1]. CP/PPS was diagnosed in 8% of all visits to urologists and 1% of all visits to primary care physicians annually in the United States [79]. Many therapeutic agents are routinely used to treat CP/CPPS including alpha-adrenergic blockers, antibiotics, and anti-inflammatory medications [80]. However, randomized controlled trials to determine the efficacy of these treatments revealed no benefit for them in the majority of patients with CP/CPPS [81,82].

OnabotulinumtoxinA has been reported to use in treating prostatitis since 1998, in that report, four male patients had improved voiding dysfunction after onabotulinumtoxinA injection [83]. In an animal study, intraprostatic onabotulinumtoxinA injection suppressed prostatic pain and inflammation in rats with capsaicin-induced prostatitis [84]. The inflammatory cell and cyclooxygenase-2 expression in the prostate significantly decreased after onabotulinumtoxinA injection. An early study in 2000 reported on 11 male patients with CP/CPPS, who received transurethral perisphincteric injection of 200 U of onabotulinumtoxinA [85]. Relief of prostatic pain and urethral hyperalgesia was observed at 2 to 4 weeks [65]. In another study, 29 male patients with CP/CPPS received 100 U of onabotulinumtoxinA or normal saline injected into the perineal body and bulbospongiosus muscle [86]. The response rate and pain score improvement were significantly better in the onabotulinumtoxinA group than in the placebo group at the 1-month follow-up evaluation.

Recently, a prospective, double-blind and randomized placebo- controlled study enrolled 60 male patients with refractory CP/CPPS to receive transurethral intraprostatic injection of 100 U of onabotulinumtoxinA or normal saline [87]. The Chronic Prostatitis Symptom Index, pain VAS, and urinary frequency significantly improved in the onabotulinumtoxinA group while none of the values improved significantly in the placebo group. The VAS score in the onabotulinumtoxinA group decreased by 62.3%, 72.4%, and 82.1% at 1, 3, and 6 months, respectively. The difference between two groups in the pain VAS score and urinary frequency differed significantly between the onabotulinumtoxinA and control groups. Another clinical trial compared intraprostatic injection of 300 U of onabotulinumtoxinA and 1% lidocaine in patients with CP/CPPS, but the study is still ongoing (ClinicalTrials.gov identifier: NCT00529386). Although clinical studies suggest onabotulinumtoxinA injection is beneficial in patients with CP/CPPS (Table 2), laboratory evidence of BoNT- A effectiveness is limited. Further studies of pain mediator release in prostate pain such as CGRP, substance P, and ATP should be investigated. Studies to compare onabotulinumtoxinA injection and other therapies in the patients with CP/CPPS are also necessary.

## 6. Clinical Application of BoNT-A in Pelvic Floor Muscle and Fascial Pain

The pelvic floor is made up of muscles and fascia, and CPP could be simply a form of myalgia. Studies show that some patients with CPP have more muscle spasm and increased muscle tone; muscle relaxation could diminish spasm and pain [88,89]. Diagnosis of pelvic floor muscle fascia and pain is made by taking a complete functional history, and a vaginal or rectal examination should be performed to assess the function of the pelvic floor muscles. Some patients also have myofascial trigger points in the puborectalis, perineum, or penis [90]. Pelvic floor pain is conventionally treated with pelvic floor muscle exercises, biofeedback, and electrostimulation [91,92]. A systematic meta- analysis suggests these treatments are effective, although high-quality evidence is still lacking [93].

OnabotulinumtoxinA was first used to treat pelvic floor spasm and pain in women in 2004. Jarvis enrolled 12 women with objective pelvic floor muscle hypertonicity and chronic pelvic pain to receive 40 U of onabotulinumtoxinA injected into the puborectalis and pubococcygeus muscles [94]. The VAS score for dyspareunia and pelvic floor muscle resting pressure 4 weeks after treatment significantly improved compared with baseline. A randomized controlled trial of 60 patients received 80 U of onabotulinumtoxinA injections or normal saline injections in the pelvic floor muscle [22]. Dyspareunia and nonmenstrual pelvic pain improved significantly in the onabotulinumtoxinA injection group, and pelvic floor pressure was also reduced (from 49 to 32 cmH2O, *p* < 0.001). In the placebo group, only dyspareunia improved significantly. The magnitude of the reduction of the pelvic floor pressure was significantly higher in the onabotulinumtoxinA group than in the placebo group. No intergroup differences for VAS pain score were found. Repeated onabotulinumtoxinA injections in female patients with pelvic floor muscle pain are reported to be as effective as the first set of injections [95].

**Table 2:** Clinical studies for the use of onabotulinumtoxin Ain chronic prostatitis/chronic pelvic pain syndrome.

Authors, year	*N*	Follow-up	BoNT-A dose	Injection sites	Results
Zermann *et al*. 2000 [85]	11	2–4 weeks	200 U	transurethral perisphincteric injection	1. relief of prostatic pain and urethral hypersensitivity/hyperalgesia
					2. decrease of the urethral sphincter closure pressure and increase maxima flow rate
Gottsch *et al*. 2011 [86]	29	1 mo	100 U or normal saline	perineal body and bulbospongiosus muscle.	1. 30% response rate for BoNT-A treatment compared with 13% for placebo (*p* = 0.0002).
					2. Pain score significantly better in BoNT-A group
Falahatkar *et al*. 2014 [87]	30	1, 3, 6 mo	100 or 200 U Normal saline	transurethral intraprostatic injection into 3 different points of each lobe	1. NIH-CPSI total and subscale scores and urinary frequency had significantly improved in BoNT-A injection, no significant improvement in placebo group
	30				2. Pain score decreased by 64.76%, 75.63%, and 79.97%

BoNT-A: botulinum toxin A.

**Table 3:** Clinical studies for the use of onabotulinumtoxin Ain pelvic floor muscle and fascia pain.

Authors, year	*N*	Follow-up	BoNT-A dose	Injection sites	Dyspareunia, Δ%	Non-menstrual pelvic pain, Δ%	Pelvic floor pressure, Δ%
Jarvis *et al.* 2004 [94]	12	4 weeks	40 U	bilaterally puborectalis and pubococcygeus muscles	−65% *	−42%	−37% *
Abbott *et al.* 2006 [22]	30	6 mo	80 U	pelvic floor muscles	−81% *	−57% *	−35% *†
	30		Normal saline		−58% *	−18%	−11% *
Nesbitt-Hawes *et al*. 2013 [95]	26	26 weeks	100 U single	puborectalis and pubococcygeous muscles	−44% *	−32% *	−17.5% *
	11		100 U repeat		−55% *		

Δ%: change from baseline, percentage. †: significant difference between different groups, BoNT-A: botulinum toxin A.

A systematic review of five studies suggested onabotulinumtoxinA injections were beneficial in relieving CPP related to pelvic floor spasm [96]. However, the others previous studies do not support onabotulinumtoxinA treatment for myofascial pain trigger points in the trunk of the body. A review article of four qualified clinical trials suggested onabotulinumtoxin Ainjections were not useful for the treatment of muscle pain trigger points in the neck or shoulders [97]. Only one study in the review article showed onabotulinumtoxin Ainjections effectively treated neck and shoulder pain [97]. For patients with pelvic floor muscle pain, the symptom improvement after onabotulinumtoxinA injection may not be the result of antinociceptive activity but might be due to the antispasticity effect on pelvic floor spasm. Although current evidence suggests onabotulinumtoxinA is an attractive option for refractory CPP related to pelvic floor muscle spasm, the limited data are preliminary. (Table 3) Additional randomized, controlled studies with large cohorts are necessary to resolve these conflicting results.

## 7. Other Applications of BoNT-A in CPP

Substance induced cystitis and radiation cystitis usually induced bladder pain in these patients, and generally definite effective treatment of these diseases did not exist. Recently, onabotulinumtoxinA has also been used for the treatment of inflammatory bladder pain diseases and showed some promise results.es. Cayan injected onabotulinumtoxin Ainto the bladders of rats with chemically induced cystitis [98]. The maximum bladder capacity and bladder compliance in the rats were significantly higher in the 2–3 U onabotulinumtoxinA intravesical injection group than in the normal saline injection group. Bladder mast cell counts and leukocyte infiltration after treatment were similar in the two groups. Chuang also reported the results of intravesical onabotulinumtoxinA treatment of six patients with refractory radiation cystitis and two patients with Bacillus Calmette-Guérin-induced chemical cystitis [24]. The CBC, urinary frequency, and VAS pain score had improved significantly in both patients with radiation and chemical cystitis 1 month following treatment. The severity of the inflammatory cell infiltration in the bladder tissue had also improved 2 months after onabotulinumtoxinA injection.

Ketamine-induced cystitis is a relatively new urological disease characterized by severe urinary frequency and bladder pain [99]. OnabotulinumtoxinA has been used to treat patients with ketamine-induced cystitis, but the efficacy is controversial. In a case series of six ketamine-induced cystitis patients that received intravesical injection of 200 U of onabotulinumtoxinA, the urinary frequency, bladder pain, and CBC had improved significantly by the 4-week follow-up evaluation [100]. However, in another case report, onabotulinumtoxinA injection was ineffective in a patient who had not quit ketamine use [101]. The failed treatment might be attributed to a chronic contracted bladder. It is possible that the bladder inflammation has come to an end stage and only augmentation cystoplasty can provide a successful treatment.

The therapeutic effect of onabotulinumtoxinA injection in these inflammatory bladder diseases might be resulted from, as above-mentioned, blocking release of noxious neurotransmitters and reducing neurogenic inflammation in bladder. However, removing the subtances which induced bladder inflammation is always the first step of treatment (for example, quit ketamine in the patients with ketamine related cystitis). Complication of BoNT-A injection such as hematuria and urinary tract infection might be more severe in the bladder with active inflammation. We suggested BoNT-A injection must be performed after subsiding of active inflammation. Furthermore, in the late stage of these inflammatory bladder disease, a contracted bladder with severe fibrosis is a common presentation. BoNT-A injection should not be effective in such bladders.

## 8. Adverse Events of BoNT-A injection in CPP

The commonest adverse event of onabotulinumtoxinA injection is urinary tract is de novo intermittent catheterization, urinary retention and asymptomatic urinary tract infection [102]. A phase 3 study of using Botox in neurogenic detrusor over activity reported 35% patient on 200 U and 42% patients on 300 U Botox developed de novo urine retention with intermittent catheterization [103]. In the aspect of using Botox in IC/BPS, Smith *et al*. reported that two of 13 IC/BPS patients developed a slow urinary stream after onabotulinumtoxinA injection [21]. Giannantoni *et al*. also reported decreased maximal urinary flow rate and detrusor pressure [67]. In a series of 67 IC/BPS patients that received 100 U of onabotulinumtoxinA, 24 (38%) experienced dysuria, but no episode of acute urinary retention developed [65]. Urinary tract infection developed in four patients (5%). In a study of 30 men with CP/CPPS, who received intraprostatic injection of onabotulinumtoxinA, only two patients complained of mild hematuria lasting less than 6 hours [87]. In terms of onabotulinumtoxinA injection in CPP due to pelvic floor muscle spasm, two women reported increased flatus, but no fecal or urinary incontinence [94]. A small amount of self-limited bleeding at the injection sites is also reported [96]. Although dysuria is the most common adverse effect in onabotulinumtoxinA treatment for CPP, no systemic complications, such as respiratory depression, muscle weakness, or fatigue are reported. The adverse events related to onabotulinumtoxinA injection in CPP are usually self-limiting. However, they should be well explained to patients before treatment. The possibility of urinary retention and necessity of intermittent catheterization also should be mentioned.

## 9. Conclusions

CPP originates from different pelvic organs, and its treatment is usually challenging. Onabotulinumtoxin A injection provides promising results in treating refractory CPP due to various pelvic organs. Current evidence suggests onabotulinumtoxin Ainjection in CPP provides effects in pain relief, and repeat injection improves inflammation in the bladder. Preliminary studies of onabotulinumtoxin Ainjection in men with CP/CPPS and pelvic floor muscle pain also showed encouraging results. Using BoNT-A to treat CPP is safe and has few self-limiting adverse effects. More randomized, placebo controlled studies in patients with IC/BPS, CP/CPPS and other conditions are necessary in order to well establish the efficacy of onabotulinumtoxinA injections in these diseases.
